# A sampling-guided unsupervised learning method to capture percolation in complex networks

**DOI:** 10.1038/s41598-022-07921-x

**Published:** 2022-03-09

**Authors:** Sayat Mimar, Gourab Ghoshal

**Affiliations:** 1 Department of Physics and Astronomy, University of Rochester, Rochester, 14627 NY USA; 2 Department of Computer Science, University of Rochester, Rochester, 14627 NY USA

**Keywords:** Complex networks, Phase transitions and critical phenomena

## Abstract

The use of machine learning methods in classical and quantum systems has led to novel techniques to classify ordered and disordered phases, as well as uncover transition points in critical phenomena. Efforts to extend these methods to dynamical processes in complex networks is a field of active research. Network-percolation, a measure of resilience and robustness to structural failures, as well as a proxy for spreading processes, has numerous applications in social, technological, and infrastructural systems. A particular challenge is to identify the existence of a percolation cluster in a network in the face of noisy data. Here, we consider bond-percolation, and introduce a sampling approach that leverages the core-periphery structure of such networks at a microscopic scale, using onion decomposition, a refined version of the *k*-core. By selecting subsets of nodes in a particular layer of the onion spectrum that follow similar trajectories in the percolation process, percolating phases can be distinguished from non-percolating ones through an unsupervised clustering method. Accuracy in the initial step is essential for extracting samples with information-rich content, that are subsequently used to predict the critical transition point through the confusion scheme, a recently introduced learning method. The method circumvents the difficulty of missing data or noisy measurements, as it allows for sampling nodes from both the core and periphery, as well as intermediate layers. We validate the effectiveness of our sampling strategy on a spectrum of synthetic network topologies, as well as on two real-word case studies: the integration time of the US domestic airport network, and the identification of the epidemic cluster of COVID-19 outbreaks in three major US states. The method proposed here allows for identifying phase transitions in empirical time-varying networks.

## Introduction

Artificial intelligence has abundant applications in a wide spectrum of disciplines including health care, medicine, finance, autonomous driving and engineering of smart devices to name a few^1^. In the physical sciences, machine learning (ML) techniques have been used to extract useful information from massive datasets generated by particle physics experiments or observations in astronomy^2^. In condensed matter physics, ML methods have been adapted to study thermodynamic phase transitions in several classical systems such as the Ising Model^3^, the *XY* Model^4,5^ and the Hubbard model^6^, as well as to explore quantum phase transitions^7–10^. On regular lattices, for instance, unsupervised learning models are used to discriminate between ferromagnetic and paramagnetic spin configurations at different temperatures, with unlabeled samples above and below the critical threshold. Examples of such methods include principle component analysis (PCA)^11^, *t*-distributed stochastic neighboring ensemble (*t*-SNE)^5^, *k*-means clustering^12^, auto-encoders^13^ and the recently introduced confusion scheme^14^. When labels with ordered-disordered states of configurations are introduced, supervised learning methods such as Artificial (ANN)^15,16^ and Convolution Neural Networks (CNN)^17^, are employed to infer the transition temperature, which has been found to be in exact agreement with theoretical predictions^18,19^

In complex networks an important example of critical phenomena is percolation, a measure of structural resilience and a benchmark model for other dynamical processes such as epidemic spreading, vital node identification and community detection^20–22^. One example is bond-percolation, where in a network of *N* nodes, *E* edges are added randomly to an empty network (or conversely removed at random from a connected network), until at a critical fraction of edges $\phi _c$, a giant connected component (GCC) of size *O*(*N*) emerges in a continuous second-order phase transition. While the critical bond-occupation probability $\phi _c$ can be computed using numerical^23^ and analytical methods^24–27^, for networks of smaller size and in real-world networks with incomplete data, due to finite size effects these differ from what can be determined by directly simulating the percolation process^28^.

Indeed, data on empirical networks is commonly noisy and restricted to sub-samples^29^. Furthermore, uncertainty in measurements alters network topology and impacts structural measures such as centrality as well as the percolation threshold, which in turn affect network dynamics^30–34^. In particular, nodes in the network-core are more sensitive to incomplete observations and missing links^35,36^. Examples include Call Data Records (CDR’s) from mobile-phones that miss connections due to missing phone numbers, as well as online social networks that may indicate existing virtual ties among people who are unacquainted^37^. In the context of epidemics, super-spreading events have been identified as a significant source and driver of major outbreaks^38^. However, contact-tracing the network of spread is biased by limitations in data collection and public health capacity, potentially leading to over- or under-estimation of the extent of super-spreading^39^.

Recently, ML techniques have been proposed to study the epidemic cluster in the susceptible-infectious-susceptible (SIS) compartmental model of epidemic spreading on networks^40^. The proposed approach converts high dimensional network data into image-like structures and exploits CNNs to learn and precisely identify the outbreak threshold of epidemic dynamics. Similar techniques were used for the case of sparse time-series data of a handful of nodes produced by networks of coupled Kuramoto oscillators^41^ to accurately classify underlying network structure. In^42^ a deep learning framework is introduced, combining both unsupervised and supervised learning methods to predict phase transitions associated with spreading dynamics. This approach makes accurate estimates of the critical transition point in uniform random networks, as well as proposes hub- and -neighbors and max-*k*-core sampling to overcome predictive inaccuracies associated with networks that have heavy-tailed distributions of links. Indeed, the rich structural features of heterogeneous networks render the simultaneous prediction of the critical transition point and the clustering of dynamical phases by adopting unsupervised learning approaches like PCA, challenging. In complex hierarchical networks, separating percolating and non-percolating regimes in dynamical processes remains unsolved.

To uncover the precise role of network structure in the learning process, in this manuscript, we focus on bond-percolation and investigate the effect of topology on ML methods that seek to estimate the percolation clusters and to infer the critical bond occupation probability $\phi _c$. Our approach extends previously proposed macro-level sampling procedures by using onion decomposition (OD)^43^ as a tool to determine the position of nodes in the core-periphery structure. This network statistic—a refined version of the *k*-core decomposition—decomposes the network into hierarchically ordered layers and reveals much more structural information at meso and micro scales. OD is able to uncover the internal structure of a *shell* (determined by *k*-core) by introducing the concept of *layer* that counts the number of peeling steps needed to reach a node, hence identifying a succession of layers within a *shell*^44^. The resulting onion spectrum obtained with this pruning method effectively defines the center and periphery of a network. We investigate the limiting cases of uniform—and heavy-tailed—distribution and demonstrate significant differences between networks of opposite topologies, with the former having a homogeneous population of nodes across layers, while the latter containing dense layers interspersed by sparse regions. We use a hybrid unsupervised learning method, combining *t*-SNE and *k*-means clustering, and train it on subsets of nodes sampled from both the sparse and dense layers to distinguish dynamical states above and below $\phi _c$. We show that sampling from the dense layers with nodes containing similar dynamical information in the percolation process, provides significantly higher accuracy than compared to sampling from the sparse layers or sampling nodes randomly, independent of whether nodes lie in the core or the periphery.

Having determined the optimal sampling strategy, we next use the confusion scheme to identify the critical occupation probability $\phi _c$, and once again demonstrate high accuracy as compared to the ground-truth estimates of the threshold values. Perhaps, most surprisingly, we show that optimal samples are not limited to the core of the underlying network, but there exists multiple subset of nodes in the entire range of layers in the onion spectrum. This finding bears particular significance for using such methods in empirical networks, the majority of which have heavy-tailed distributions of links, and whose measurements are noisy. Finally, we apply our formalism to two examples of real-world time evolving systems: We determine the exact critical integration time of the US air transportation network , as well as identify the epidemic cluster for COVID-19 in three major states in the US. We end with a discussion of the implications of our findings.

## Percolation on different network topologies

Consider a network *G* where $\mathcal {V}=\left\{ v_{1}, \cdots , v_{N}\right\}$ is the node set that undergoes bond-percolation, and let $\phi$ denote the occupation probability of a certain configuration. The data $\mathbf{X}$ generated during the process is contained in a $M \times N$ matrix, where *N* is the number of nodes and *M* is the total number of dynamical states at different values of the control paramete $\phi$. The entries of $\mathbf{X}$ are binary;

1 $$\begin{aligned} x_{(v_i, \phi )}=\left\{ \begin{array}{ll} 1, &{} v_i \in GCC \\ 0, &{}v_i \notin GCC. \end{array}\right. \end{aligned}$$

Each value is characterized by the tuple $(v_i, \phi )$ and equals 1 if $v_i$ is part of the giant connected component (GCC) of the network and 0 if the node is disconnected from GCC at given $\phi$. The matrix **X** is then associated with the vector **y** of size $M \times 1$ that represents the label space. For pre-transition states $\phi \ge \phi _c$ the entries $y(\phi ) = 0$ and for post transition states $\phi < \phi _c$ we have $y(\phi ) = 1$.

We pick two graphs at the opposite ends of the structural spectrum of networks: first, we construct a square lattice with $N^{SL} = 1024$ and open boundaries such that all nodes, except those at the periphery, have degree $k=4$, leading to a uniform, tightly-peaked degree distribution. On the other end of the spectrum, we consider a power-law network with $N^{PL} = 1000$ and degree distribution $p_k \sim k^{-\gamma }$ generated using the configuration model^45^. We choose an exponent $\gamma = 3.1$, such that both networks have finite second moments $\langle k^2 \rangle$ in their degree-distributions and exhibit phase transitions at non-zero probabilities $\phi _c = \left[ \langle k^2 \rangle /\langle k \rangle -1\right] ^{-1}$ in the thermodynamic limit^24^. We simulate the percolation process on the networks with opposite topologies starting from $\phi = 1$ and decrementing the occupation probability by $\Delta \phi = 0.001$ until $\phi = 0$ yielding $M = 1000$ configurations in total for both networks, generating the matrices $\mathbf{X} ^{SL}$ with size $1000 \times 1024$ and $\mathbf{X} ^{PL}$ with size $1000 \times 1000$ for square lattice and power-law network respectively. The critical phase transition point $\phi _c$ is numerically determined as the value of $\phi$ where the size of the second largest cluster reaches its maximum^46^. The ground truth label vectors $\mathbf{y} ^{SL}$ and $\mathbf{y} ^{PL}$ with entries either 0 or 1 representing two percolating classes are constructed accordingly. Hence the pairs $\{ \mathbf{X} ^{SL} : \mathbf{y} ^{SL} \}$ and $\{ \mathbf{X} ^{PL} : \mathbf{y} ^{PL} \}$ contain the training data and corresponding labels that are used in the subsequent machine learning methods described in next sections.

Next, we use the onion decomposition method to uncover the core-periphery structure of two different types of networks. In addition to the *coreness* metric produced by *k*-core decomposition that identifies nested maximal subnetworks with nodes having at least *k* connections, the onion decomposition improves the *coreness* information by assigning a $\textit{layer}$ to each node, to further indicate its position within the core and makes its internal organization apparent^43^. (See SI Sect. 1 for the details of OD algorithm and demonstration of the method on a sample network in Fig. S1).

**Figure 1:**
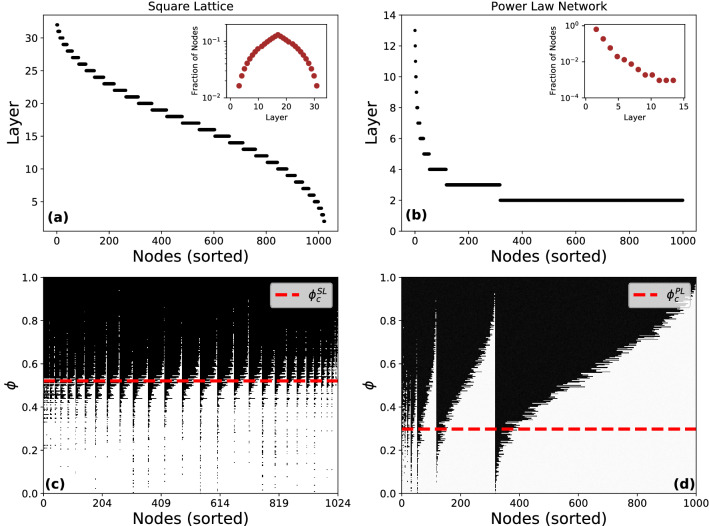
Effect of topology on percolation dynamics. Onion decomposition of (**a**) a Square Lattice of size $N^{SL} = 1024~(32\times 32)$ and (**b**) a power-law network with $N^{PL}=1000$ and $\gamma = 3.1$. Nodes are ordered from the inner- to the outer-most layers which are labeled in descending order. The onion spectra are shown in the inset, as distribution of nodes per layer, where 32 and 14 distinct layers are identified by square lattice and power law network, respectively. Layers are populated uniformly in the square-lattice, and in a punctuated fashion. In (**c**) and (**d**), the percolation process in the range $0 \le \phi \le 1$ represented by the matrices $\mathbf{X} ^{SL}$ and $\mathbf{X} ^{PL}$ for the square-lattice and power-law network where nodes (columns) are sorted in decreasing order with respect to their layer values from core to periphery (left to right). The critical bond-occupation probabilities $\phi _c^{SL} = 0.524$ and $\phi _c^{PL} = 0.298$ are marked as the red dashed line. Nodes part of the GCC are colored black, those outside are colored white. Nodes are ordered the same as in the upper-panel.

In Fig. 1a,b we show the onion spectra of the square lattice and the power-law network. Nodes are sorted with respect to their $\textit{layer}$ value in descending order, from the inner- to the outer-most layer—from core to periphery—(high to low $\textit{layer}$ values from left to right). The square lattice has a uniform spectrum where nodes populate each layer equally in the network, however the power-law network shows a spectrum with sparsely filled inner layer followed by monotonically increasing node fractions in successive layers as one moves to the outer layers, terminating in a large peripheral layer. The fraction of nodes in each layer (shown as inset in both panels) indicates that square-lattice and power law network have 32 and 14 onion layers in their onion spectra as opposed to a single and double shells respectively, identified by *k*-core decomposition. The effect of this difference in structure is shown in panels c and d, where we show the evolution of the percolation process in the range $0 \le \phi \le 1$ by plotting the matrices $\mathbf{X} ^{SL}$ and $\mathbf{X} ^{PL}$ where nodes (columns) are sorted with respect to their layer values in decreasing order from left to right and the horizontal axis corresponds to the nodes sorted in the same order as in the upper panel. The critical bond occupation probabilities $\phi _c^{SL}$ and $\phi _c^{PL}$, below which there is no GCC, are marked by the red horizontal dashed lines. Nodes that are part of the GCC are colored black, whereas those outside the GCC are colored white. (Note that the few sets of nodes colored black below $\phi _c$ belong to the largest connected component (LCC) which is technically not the GCC.).

The figure indicates that in the case of the square lattice, groups of nodes across layers show common dynamics for a wide-range of $\phi$. Nodes attach and detach from the GCC (as $\phi$ changes) in a similar fashion independent of what layer they belong to. In contrast, in the power-law network there is wide variation across layers; for a given value of $\phi$ large swathes of nodes in the inner- and outer-most layers are not part of the GCC. Further, whether a node is part of the GCC as $\phi$ is increased, varies from layer-to-layer. Finally, we note the presence of high-fidelity samples in the core-, intermediate- and peripheral-layers. For any classification algorithm, such class-imbalanced datasets pose a challenge as learning methods fail to capture the distributive characteristics of the data and produces unsatisfactory accuracies^47^. The performance of such algorithms is poor on subsets with under and over-represented classes as it tends to partition phases into relatively uniform sizes. The pre-processing of the data using onion decomposition, instead allows for the identification of node subsets with similar dynamical information in the percolation process. That is, the method identifies layers where nodes disconnect from the GCC at comparable values of the control parameter, hence yielding a balanced training data in the subsequent learning phase and leading to high clustering accuracy of dynamical phases. This is particularly important for the power law network as shown in Fig. 1b where the fractions of nodes present in different layers and their corresponding dynamical trajectories panel (Fig. 1d) display an irregular trend.

## Effect of sampling on clustering

Next, we focus on the classification scheme for clustering configurations as percolating and non percolating phases. We construct a hybrid unsupervised learning model with *t*-SNE^48^ a non-linear dimensionality reduction technique, and *k*-means clustering^49^ used for identifying pre-determined number of clusters from an unlabeled dataset. We add Gaussian noise, $\mathcal {N}(0,\,0.01)$, to the matrices $\mathbf{X} ^{SL}$ and $\mathbf{X} ^{PL}$ with sorted columns (1$^{\mathrm{st}}$ column being the node in innermost layer) to help spread the data points. We then sample subsets of nodes in consecutive disjoint bins of size 20 in the form of 1–20, 21–40, 41–60, etc. (corresponding to $\approx 50$ samples of $1000 \times 20$ matrices, covering the entire range of nodes) as training sets, from the innermost layer to the peripheral layer such that nodes with similar layer values are grouped into a single sample. We project subsets of 20 nodes into a two-dimensional plane with *t*-SNE, and then use *k*-means with $k = 2$ to assign pre and post transition labels to all 1000 configurations in hand. To assess the performance of the algorithm we compare the labels assigned by the unsupervised learning method $\varvec{{\hat{y}}}$ to the ground-truth labels $\mathbf{y}$ for both square lattice and power law network and define the accuracy $\alpha _{{\hat{y}}, y}$ as

2 $$\begin{aligned} \alpha _{{\hat{y}}, y}=\frac{1}{n_{\text{states }}} \sum _{i=1}^{n_{\text{states }}} \delta _{{\hat{y}}, y}, \end{aligned}$$

where the summand is the Kronecker delta-function.

**Figure 2:**
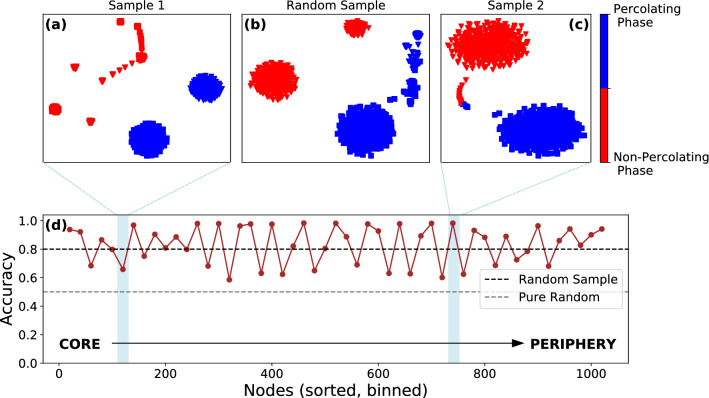
Effect of sampling on the unsupervised learning scheme for the square lattice. (**d**) The accuracy of label prediction $\alpha _{{\hat{y}}, y}$ with *t*-SNE and *k*-means clustering as a function of training data consisting of binned nodes of size 20, sorted in decreasing order with respect to their layer values from core to periphery as shown by the arrow from left to right. The accuracy for random samples of 20 nodes ($\alpha _{{\hat{y}}, y} = 0.8$) is shown as a black dashed line, and the grey dashed line corresponds to model-independent random guessing of state labels ($\alpha _{{\hat{y}}, y} = 0.5$). Examples of clustering of percolating (blue dots) and non-percolating phases (red dots) (**a**) for low-accuracy samples from the inner-layer (**b**) random samples and (**c**) high-accuracy samples from the outer-layer.

In Fig. 2 we plot the results of our analysis for the square-lattice. Panel d shows $\alpha _{{\hat{y}}, y}$ as a function of the sampled subsets of nodes sorted with respect to their layer values. As baselines, the horizontal black dashed line indicates the accuracy for 50 samples of subsets of 20 nodes chosen uniformly at random independent of layer position ($\alpha _{{\hat{y}}, y} = 0.80$) and the gray dashed line represents the accuracy for a model-independent random guess of the state-labels ($\alpha _{{\hat{y}}, y} =0.5$). As the figure indicates, depending on the sampled layer, accuracy fluctuates around the performance of the random sampling method, with some layers providing almost perfect accuracy, whereas others no better than a random guess of labels. The high-and low-accuracy samples are not limited to the core, but periodically found across all layers in the onion spectrum. Example outputs of the unsupervised learning scheme are shown in panel a (inner-layer, $\alpha _{{\hat{y}}, y} =0.56$), b (random sample, $\alpha _{{\hat{y}}, y} =0.80$) and **c** (outer-layer, $\alpha _{{\hat{y}}, y} =0.99$). For the poor-accuracy inner-layer sample, the model is confused by the fact that nodes in the training-set exhibit different dynamical evolution in the percolation process. They connect to the GCC at different values of $\phi$, hence the unsupervised learning model cannot divide configurations into two separate clusters. Conversely, for the high-accuracy outer-layer sample, the subset of nodes belongs to a high-fidelity layer in the onion spectrum, such that the majority of nodes in the set connect to the GCC at similar values of $\phi$. Such layers, as indicated in Fig. 1c are distributed equally across the onion spectrum. Finally, the random sampling strategy provides reasonable accuracy, given the homogenous structure of the square lattice (all nodes in a single-shell), as random samples and tailored subsets have similar properties.

**Figure 3:**
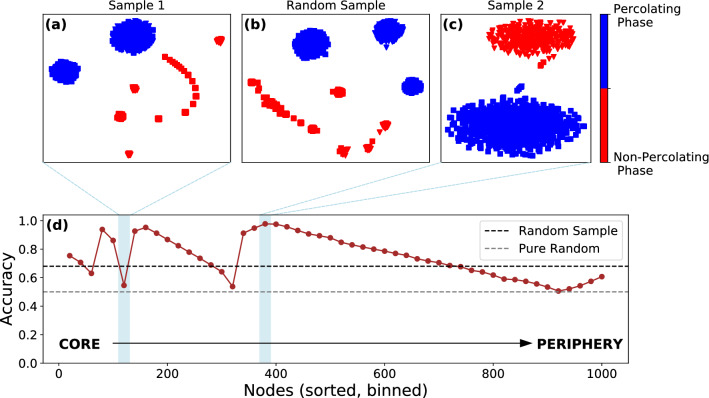
Effect of sampling on the unsupervised learning scheme for the power-law network. (**d**) The accuracy of label prediction $\alpha _{{\hat{y}}, y}$ with *t*-SNE and *k*-means clustering as a function of training data consisting of binned nodes of size 20, sorted in decreasing order with respect to their layer values from core to periphery as shown by the arrow from left to right. The accuracy for random samples of 20 nodes ($\alpha _{{\hat{y}}, y} = 0.64$) is shown as a black dashed line, and the grey dashed line corresponds to model-independent random guessing of state labels ($\alpha _{{\hat{y}}, y} = 0.5$). Examples of clustering of percolating (blue dots) and non-percolating phases (red dots) (**a**) for low-accuracy samples from the inner-layer (**b**) random samples and (**c**) high-accuracy samples from the outer-layer.

In Fig. 3 we plot the corresponding results for the power-law network indicating rather different behavior. As seen in panel b, the random sampling (averaged over $\sim 50$ random subsets) strategy performs considerably poorer with $\alpha _{{\hat{y}}, y} = 0 .64$, only marginally better than randomly guessing labels. Furthermore, the peaks and troughs in the accuracy curve are much more irregular, as compared to the square-lattice, reflecting the richer structure of the power-law network. There are samples in the inner and intermediate-layers providing poor accuracy (panel a), and surprisingly, there exists a range in the outer-most layers that yields accuracy as high as 0.99 (panel d). The results indicate the importance of adopting a considered sampling strategy for training-sets in power-law topologies, given that unlike in networks with uniform topologies, random sampling is sub-optimal. Indeed, very few real-world networks have uniform topologies, instead exhibiting heavy-tailed distributions, implying that for any realistic application, identifying high-quality samples a priori is of paramount importance. Given issues of data sparsity, it is of note, that such samples exist in multiple layers of the power-law network, including the core-, intermediate- and peripheral layers.

## Identifying critical transition points

The procedure described thus far, while effective in identifying configurations below and above the percolation phase, in itself, cannot identify the critical bond occupation probability $\phi _c$. To do so, we make use of the confusion scheme, first introduced to study phase transitions in Kitaev chains, the classical Ising model and in disordered quantum spin chains^14^. Recently it has been extended to uncover the critical transition probability in dynamical phase transitions in complex networks^42^. Next, we show that our sampling-guided strategy adopted to the confusion scheme is quite effective in terms of identifying the value of $\phi _c$.

The method does not take as input labels of the dynamical states, instead a synthetic label space $\mathbf{y}$ is associated with an input matrix $\mathbf{I}$, with entires 0 and 1, corresponding to the before and after transition states in the percolation process. At $\phi = 1$ the label vector $\mathbf{y} = \mathbf{0}$ i.e., all configurations are in the before state, and for $\phi = 0$, the vector $\mathbf{y} = \mathbf{1}$, each snapshot is labeled as after state. The boundary between 0 and 1 (corresponding to the critical threshold) in this artificial label-set is varied in the entire range of the control parameter $\phi \in [0,1]$ with increments of $\Delta = 0.005$ (yielding 200 steps in total) and associated with an optimal subsample $\mathbf{I} \subset \mathbf{X}$ selected using the method described in “Effect of sampling on clustering”. For both the square-lattice and the power-law network we select samples from the periphery that yield high accuracy in clustering.

A feed-forward neural network (FFNN) is trained with this data consisting of pairs $\{\mathbf {I}: \mathbf {y} \}$ in the form of supervised learning problem using the PyTorch library^50^. The input layer contains neurons at the same number of the chosen subsample size, followed by a hidden layer of 128 neurons. Both layers have rectified linear unit (ReLu) as activation functions. The output layer contains a single neuron with sigmoid activation function, that predicts the probability of a configuration belonging to one of the states. A binary cross-entropy loss-function is minimized in training, which is well suited for binary classification problems. For stochastic optimization we use the Adam method^51^ with learning rate $10^{-3}$. To prevent over-fitting we use Dropout regularization with probability $10^{-1}$. In each step, the dataset is split into a training set that is fed into the FFNN and prediction accuracy is evaluated on the test set. Highest accuracies are achieved at endpoints of the threshold range due to the constant nature of the label space. As one spans the range between [0, 1], initially lower accuracy values are observed as some configurations are associated with incorrect labels. At the transition probability, the artificial label space matches the ground truth, leading to a high classification accuracy. In this process, the accuracy curve follows a **W**-shape as a function of $\phi$, where the middle peak corresponds to the estimated transition probability^42^ ( See SI Sect. 2 for detailed information about the confusion scheme and a schematic illustration of the output in Fig. S2).

**Figure 4:**
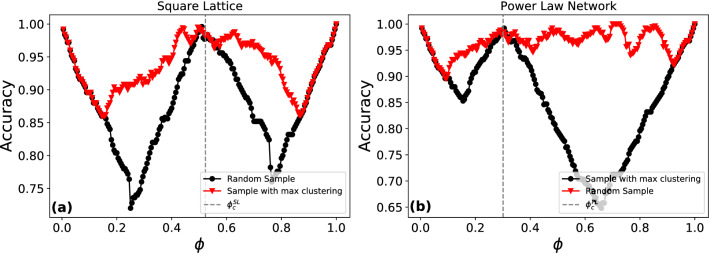
Identification of $\phi _c$ as a function of network topology: (**a**) Square lattice and (**b**) power-law network with random samples of 20 nodes (brown curve) and a high-accuracy sample from the peripheral layers identified using the unsupervised learning method (black curve). The vertical dashed line represents the critical bond occupation probabilities in each network, $\phi _c^{SL} = 0.524$ and $\phi _c^{PL} = 0.298$. The random sample in the square lattice has a clear **W** shape, but the middle peak is noisy and is flat around $\phi _c^{SL}$. For the power-law network the random sampling strategy fails to provide any information on the transition probability. In both cases, the sampling-guided strategy yields high accuracy on $\phi _c^{SL,PL}$, with the middle peaks in the black curves occurring at 0.520 and 0.305.

In Fig. 4, we show the output of the confusion scheme on the square lattice (a) and the power-law network (b). In both panels, the brown curve corresponds to a random sample of 20 nodes and the black curve to the high-accuracy subset from the peripheral layers, identified using the unsupervised learning method. The vertical dashed lines mark the value for the ground-truth value of $\phi _c$ in each network. The random sampling strategy in the square lattice is reasonably effective, generating a $\mathbf{W}$-shape, although the peak near $\phi _c$ is not well-defined. In the power-law network the random sampling accuracy curve is noisy and flat yielding little-to-no information on the transition probabilities. However, in both cases the black curve yields a clear $\mathbf{W}$-shape and the middle-peaks line up well with the ground-truth values of $\phi _c$. Thus unlike existing methods, the sampling-guided scheme outlined here simultaneously identifies nodes in the GCC as well as provides accurate estimates for the bond-occupation probability.

## Transitions in time-varying real-world networks

Next, we validate our methodology in two real-world examples. The availability of large time-resolved datasets enables for the representation of a wide range of dynamical phenomena in the form of time-varying networks. Such processes often exhibit phase transitions, and thus can be analyzed via percolation in static networks. Some applications include wireless communication networks with unreliable links^52^, spreading of infectious diseases in modular time varying networks^53^ or percolation in ground-transportation networks to identify critical bottleneck roads in local flows^54,55^. Recently, the integration process of air traffic into a temporally connected network was modeled as a time-varying percolation process^56^. The critical integration time $T_c$, at which the network forms a temporal spanning cluster, is proposed as a measure of global reliability of air-traffic.

We test our scheme on the air-transportation network, to identify both $T_c$ as well as label nodes that belong to the time-varying GCC. We construct the temporal air-transportation network^57^ starting from $t_0 = 7:30$ a.m. on September 5, 2019 and spanning a 18-h period until the integration process is completed. The resulting network consists of 288 airports as nodes and 1903 edges, where a link corresponds to at least one flight between two airports. We generate states with a time window of $\left[ t_{0}, t_{0}+T\right]$ where *T* is incremented in intervals of 1-min generating $\sim$1100 instances as the training and test set.

**Figure 5:**
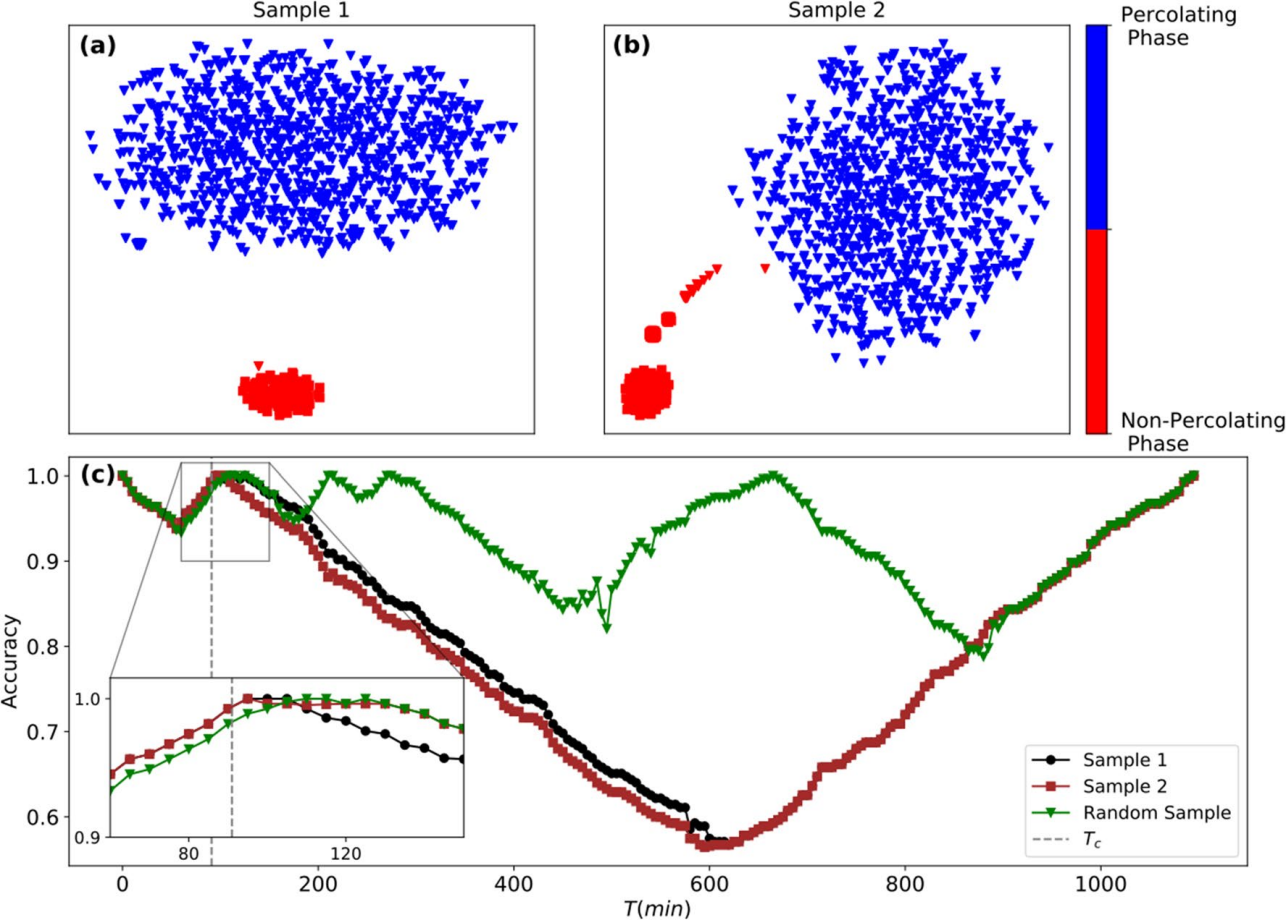
Predicting integration time of the Unites States air-transportation network. Clustering of pre- and post-transition states in the temporal integration process from a sample taken from the inner-layer (Sample 1) (**a**) and one from the peripheral layer (Sample 2) (**b**). In both cases the accuracy is $\alpha _{{\hat{y}}, y} = 0.99$. In (**c**), the output of the confusion scheme in predicting the critical integration time ($T_c = 91$ min, marked as vertical dashed line) at which a connected percolating cluster of airports linked by flights is formed. Training the network on Sample 1 yields a peak of the **W**-shaped curve (black-circles) at $T = 93$ min and with Sample 2 (red squares), at $T = 94$ min. Training on a random sample (green triangles) yields a noisy curve, and an accurate identification of $T_c$ is not possible (Behavior of all three curves near $T_c$ shown as zoomed inset).

The task of the unsupervised learning method is to discriminate between states before and after the integration process. Unlike in the synthetic networks studied thus far, the transition happens at an early stage ($T_c = 91$ min), and therefore the dataset is unbalanced. After running the onion-decomposition scheme to identify the layers, we pick two samples with 10 airports: Sample 1 from the core of the air transportation network *(Atlanta, Austin, Nashville, Boston, Charlotte, Denver, Detroit, Fort Lauderdale-Hollywood, Las Vegas, Los Angeles, Chicago O’Hare)* and Sample 2 from the periphery *(Norfolk, Worcester, Southwest Oregon, Barkley, Palm Beach, Hilton Head, Punta Gorda, Pitt-Greenville, Newport News/Williamsburg, Ithaca Tompkins)*. We then use the *t*-SNE method to cluster the states. The results are shown in Fig. 5a,b, indicating that the method performs well; the labels are assigned by *k*-means with an accuracy of $\alpha _{{\hat{y}},y} = 0.99$ in both samples of nodes. We then use these two samples as a training set on the confusion scheme, and plot the resulting accuracy curve as a function of the time *t* in Fig. 5c. The curve corresponding to the core-sample is shown as black circles, while curve for the peripheral sample is shown as red-squares. As a reference we show the case for a random sample of 10 airports *(Reno-Tahoe, Albany, Rapid City Regional, Hector, Miami, Waterloo, Kansas City, Corpus Christi, Columbia Metropolitan, Baltimore/Washington)* as green triangles, whereas $T_c$ is shown as the vertical dashed line. For both samples, the accuracy curve $\alpha _{{\hat{y}}, y}$ shows a clear $\mathbf{W}$-shape with peaks at $T= 93$ min and $T= 94$ min. The random sample yields a noisy curve with no clear peak (behavior near peak for all three curves shown as inset). The results illustrate the versatility of the sampling-scheme with near-perfect identification of airports in the temporally connected cluster and the ability to estimate the critical integration time to within 3–4$\%$, and the flexibility in sampling from the core or the periphery of the network.

**Figure 6:**
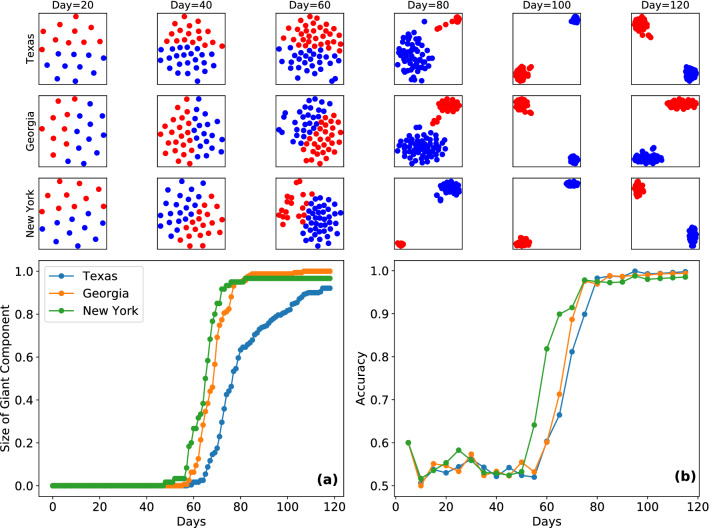
Emergence of the epidemic cluster of COVID-19 in 3 major US states. The network corresponds to population flows between locations in-state at the resolution of counties. Nodes are labeled as infected if the number of cases per-capita exceeds $10^{-4}$. (**a**) indicates that an epidemic cluster emerges around day 60 in all three states. The top three rows show the output of the clustering method that splits counties into those belonging to the epidemic cluster and those outside. Training occurs from day 0 in cumulative intervals of 20. The identification of counties belonging to the epidemic cluster becomes increasingly accurate past the phase-transition and peaks around day 80 as seen in (**b**).

Next, we consider a different dynamical process of particular relevance; the spread of COVID-19 in the United States^58^. We investigate the possibility of employing our clustering method to use as a diagnostic tool that signals at a relatively early stage, whether an epidemic outbreak is about to occur based on real-time data. We pick three major states; Texas, Georgia and New York with 3.0 , 1.1 and 1.9 ($\times 10^7$) inhabitants respectively. We consider a spatial resolution at the level of counties leading to 254 nodes for Texas, 159 for Georgia and 62 for New York. We construct mobility networks from the United States census bureau’s LODES^59^ commuting data, where the edges represent population-flows between counties corresponding to 20,262 (Texas), 11,042 (Georgia) and 1883 (New York) undirected links. We then follow the temporal evolution of the number of detected cases in each county from January 21st to May 18th 2020^60^. Counties are labeled “infected” when the number of cases per-capita is above a threshold of $10^{-4}$. In Fig. 6a, we plot the empirical temporal evolution of the number of infected counties finding an emergence of an epidemic cluster around day 60 for all three states. In the top-row of Fig. 6, we train our unsupervised learning model with samples of size 10 selected from the high-fidelity layers of of the mobility networks in cumulative intervals of 20 days starting from day 0. In Fig. 6b we plot the accompanying accuracy curve $\alpha _{{\hat{y}}, y}$ in function of time. Before day 60, there is no epidemic cluster, and therefor $\alpha _{{\hat{y}}, y} = 0.5$ equivalent to model-independent simple guessing of labels. After day 60, however, once an epidemic cluster emerges, the model is able to reliably split counties into infected and disease-free clusters and make more accurate predictions. The increase in the accuracy curve tracks the growth in the epidemic cluster and reaches perfect accuracy at around day 80. We note that from a point of forecasting the epidemic progression in real-time with up-to-date data, the model reaches accuracies of $\approx 70\%$ when the size of the epidemic cluster is $\approx 0.4$.

## Discussion

Taken together, our work sheds light on the role of micro- and mesoscopic structure of networks in machine learning phases of bond percolation. Our sampling guided approach reveals the importance of choosing particular subset of nodes from layers of the onion spectrum of the network that enables unsupervised learning methods to distinguish between percolating and non-percolating states. Labels assigned by *k*-means match the ground truth labels with near-perfect accuracy. We show that this is facilitated by sampling nodes chosen from both core and peripheral layers, identified using onion decomposition, that identifies subsets of nodes in a spectrum of layers following similar paths in the percolation process, i.e. they detach (or attach) to the percolating cluster at comparable values of $\phi$. The sampling-guided strategy carries over to other learning tasks, such as identifying the critical occupation probability $\phi _c$ using the confusion scheme. This gain in performance is particularly pronounced for networks with heavy-tailed degree distributions, where the method significantly outperforms random sampling. Indeed, to the best of our knowledge, the framework presented here is the first to simultaneously enable the clustering of nodes into different dynamical states, as well as identify $\phi _c$ in networks with heterogeneous topologies. This bears significance, given that many empirical networks exhibit right-skewed distributions of links.

To validate our results, we demonstrate two possible applications of our findings on real-world time-varying networks that exhibit a percolation transition; the exact integration time of the US domestic air transportation network, as well as the emergence of the COVID-19 epidemic cluster in three large US states. In both cases the framework yields excellent performance. The application to pandemic settings is of particular interest, as a possible diagnostic tool to assess the current state of disease-spread with real-time data. The ability to accurately classify (with reasonable accuracy) regions into infected and disease-free states (close to when the epidemic cluster first emerges) could prove useful in terms of mitigation strategies. Indeed, techniques have been proposed to study immunization strategies in networks where only a small subset of nodes are observed at a time, to slow-down epidemic spread^61,62^. Given the limited knowledge of network structure, immunizing a small sample of nodes provides significant improvement in the global level immunization of the network^63^. Similar considerations apply in the diffusion of rumors or “fake news” in social media and online platforms^64^. The approach proposed here can, in principle, be easily extended to such real world dynamical processes on networks.

## Supplementary information

Supplementary Information.
