# Supplementary material for: A sampling-guided unsupervised learning method to capture percolation in complex networks
Source: Sci Rep. 2022 Mar 9;12:4147. doi: 10.1038/s41598-022-07921-x (PMC8907239; doi:10.1038/s41598-022-07921-x)
Supplement: Supplementary file 1 — Supplementary Information. [file 41598_2022_7921_MOESM1_ESM.pdf]

Supporting Information

A sampling-guided unsupervised learning method to capture percolation in complex networks

S. Mimar and G. Ghoshal

Table of Contents

S1 Onion Decomposition **2**

S2 Confusion Scheme **3**

List of Figures

S1 Schematic representation of Onion Decomposition for a sample network. 2

S2 An example of confusion scheme output that detects a phase transition and identifies the transition point. 3

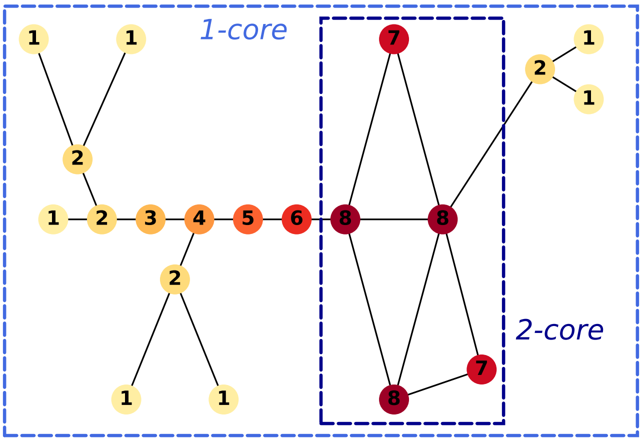

Figure S1: **Schematic representation of Onion Decomposition for a sample network.** OD starts by removing nodes with degree $k = 1$ (layer 1) and then subsequently other nodes with $k = 1$ after the initial pruning (layer 2). Iterative application of this leads to identification of layers $(3 - 6)$. The $k-$core method instead groups all of these nodes into a $1-$core and the rest into a $2-$core. Applying OD on the $2-$core leads to the identification of two more layers $7 - 8$. Node labels indicate layer numbers and colors from light to dark represent outer to inner layers in the onion spectrum.

S1 Onion Decomposition

The network pruning method, $k-$core decomposition [1] identifies subsets of the network, called $k-$cores, that are obtained by recursively deleting nodes with degree less than k until all remaining nodes have k links or more. Larger values of k indicate more connections, and hence a more central position in the network. Usign this method, one can decompose the network into hierarchically ordered shells.

The recently proposed onion decomposition method improves upon the $k-$core method, by proposing the concept of *layers* [2]. The layer values are the counts of number of node removals that are needed to reach a particular node, and reveals the hierarchical organization of each shell identified by $k-$core. An example of this method is shown in Fig. S1. The algorithm first removes the nodes with degree 1 that are located at the peripheries and assigns the layer number 1. Then, it removes nodes with 1 link (after deletion) that have layer value 2. Applying this process iteratively identifies layers $3 - 6$ until no nodes with degree 1 remains. The $k-$core method in contrast identifies all these layers as a single first core. Applying the method to nodes with at least two links leads to identification of layers $7 - 8$ within the second $k-$core. In the end, while the $k-$core method identifies two shells, the OD method uncovers eight layers, hence providing more granular information on the mescoscopic structure of the network.

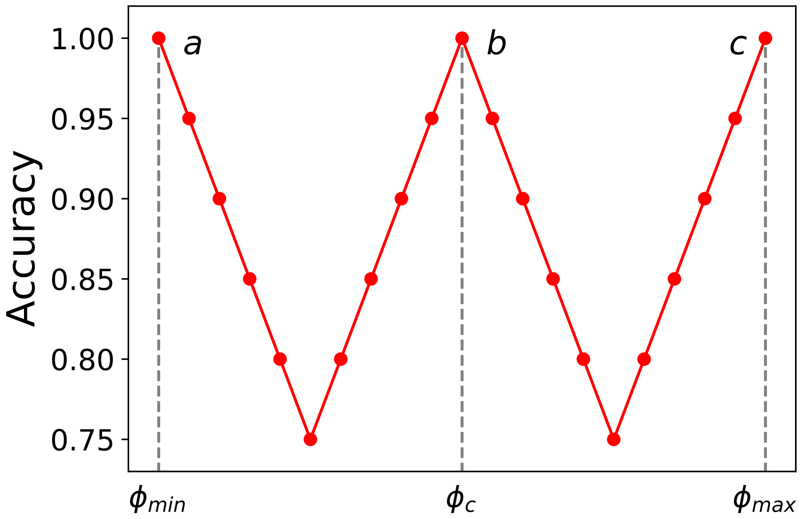

Figure S2: **An example of confusion scheme output that detects a phase transition and identifies the transition point.** The method assign tentative threshold values ϕ'_c starting from ϕ_{min} and spans the entire range of the threshold values until ϕ_{max} where there is a true critical value $\phi_c \in [\phi_{min}, \phi_{max}]$. The neural network is trained as a supervised learning method for each instance of the threshold value. Resulting accuracy curve platted with respect to the control parameter exhibit a **W** shape curve where the middle peak corresponds to the critical transition value ϕ_c.

S2 Confusion Scheme

The Confusion scheme finds the critical transition point ϕ_c in a given range of the control parameter ϕ by incorrectly labeling dynamical phases with respect to an artificial transition point ϕ'_c. As shown in Fig. S2, the method assigns labels 1 to all configurations at the threshold values $\phi'_c = \phi_{min}$ and $\phi'_c = \phi_{max}$; the neural network, trained via supervised learning, yields perfect accuracy of 100% at the peaks a and c due to the uniform nature of labels. The accuracy then reduces when the threshold value is in the ranges $\phi_{\text{min}} < \phi'_c < \phi_c$ and $\phi_c < \phi'_c < \phi_{\text{max}}$ as the neural network is "confused": some dynamical configurations are incorrectly labeled, leading to a decrease in accuracy values. When the true transition value is reached $\phi'_c = \phi_c$, the artificial or "fake" labels perfectly overlaps with the correct ones and yields a high accuracy value again at the peak c. The method indicates the existence of a phase transition in a given range $[\phi_{\text{min}}, \phi_{\text{max}}]$ of the control parameter and determines the transition value, without requiring the ground truth dynamical phase labels.

References

[1] Batagelj, V. & Zaveršnik, M. Fast algorithms for determining (generalized) core groups in social networks. *Advances in Data Analysis and Classification* **5**, 129–145 (2010). URL `https://doi.org/10.1007/s11634-010-0079-y`.

[2] Hébert-Dufresne, L., Grochow, J. A. & Allard, A. Multi-scale structure and topological anomaly detection via a new network statistic: The onion decomposition. *Scientific Reports* **6**, 31708 (2016). 1510.08542.
